# Perovskite-structured CaTiO_3_ coupled with g-C_3_N_4_ as a heterojunction photocatalyst for organic pollutant degradation

**DOI:** 10.3762/bjnano.9.62

**Published:** 2018-02-21

**Authors:** Ashish Kumar, Christian Schuerings, Suneel Kumar, Ajay Kumar, Venkata Krishnan

**Affiliations:** 1 School of Basic Sciences and Advanced Materials Research Center, Indian Institute of Technology Mandi, Kamand, Mandi 175005, Himachal Pradesh, India

**Keywords:** CaTiO_3_, graphitic carbon nitride (g-C_3_N_4_), heterojunction photocatalyst, pollutant degradation

## Abstract

A novel graphitic carbon nitride (g-C_3_N_4_)–CaTiO_3_ (CTCN) organic–inorganic heterojunction photocatalyst was synthesized by a facile mixing method, resulting in the deposition of CaTiO_3_ (CT) nanoflakes onto the surface of g-C_3_N_4_ nanosheets. The photocatalytic activity of the as-synthesized heterojunction (along with the controls) was evaluated by studying the degradation of an aqueous solution of rhodamine B (RhB) under UV, visible and natural sunlight irradiation. The CTCN heterojunction with 1:1 ratio of g-C_3_N_4_/CT showed the highest photocatalytic activity under sunlight irradiation and was also demonstrated to be effective for the degradation of a colorless, non-photosensitizing pollutant, bisphenol A (BPA). The superior photocatalytic performance of the CTCN heterojunction could be attributed to the appropriate band positions, close interfacial contact between the constituents and extended light absorption (both UV and visible region), all of which greatly facilitate the transfer of photogenerated charges across the heterojunction and inhibit their fast recombination. In addition, the two-dimensional (2D) morphology of g-C_3_N_4_nanosheets and CT nanoflakes provides enough reaction sites due to their larger surface area and enhances the overall photocatalytic activity. Furthermore, the active species trapping experiments validate the major role played by superoxide radicals (O_2_^−•^) in the degradation of pollutants. Based on scavenger studies and theoretically calculated band positions, a plausible mechanism for the photocatalytic degradation of pollutants has been proposed and discussed.

## Introduction

Photocatalysis is recognized as an attractive approach for environmental remediation and energy generation applications due to its potential towards utilization of solar energy [1–3]. Therefore, extensive efforts have been made for the design and synthesis of highly efficient and stable photocatalyst systems over the past few decades [4–7]. Hence, the development of such photocatalysts with high stability and good activity is desirable. The physical and chemical properties of a photocatalyst are related to its photocatalytic performance as three crucial factors of photocatalysis (i.e., photon absorption, charge carrier transfer and catalytic surface reactions) are dependent on them [8–9]. Two-dimensional (2D) layered heterojunctions are of great interest in the photocatalysis domain for achieving environmental sustainability [10–11]. These nanocomposites are known for their greater photogenerated electron–hole mobility across the heterojunction interface, which reduces the distance and time of charge transport to impede their recombination rate [12–13]. The 2D–2D heterojunctions exhibit larger face-to-face contact area as compared with the line-to-face contact (1D–2D) and point-to-face contact (0D–2D) photocatalysts, which is responsible for their enhanced photocatalytic activity [14]. It is worth mentioning here that 2D–2D nanocomposites could effectively improve the specific surface area and provide abundant reaction sites to adsorb reactant species on their surface, which can significantly enhance the photocatalytic activity [15–16].

Recently, graphitic carbon nitride (g-C_3_N_4_), which is a metal-free polymeric organic semiconductor with tri-s-triazine units, has drawn huge attention from researchers due to its excellent photocatalytic performance and unique properties such as appropriate band structure, visible light absorption and high chemical and thermal stability [2,4]. In addition, g-C_3_N_4_ consists of earth-abundant carbon and nitrogen elements and the low cost of the initial precursors promotes it as a promising photocatalytic material for diverse applications, such as energy generation [17–19], sensor [20], and fuel cell applications [21], as well as environmental remediation [22]. The band gap of g-C_3_N_4_ lies between 2.4–2.8 eV and therefore it has the capability to absorb visible light irradiation [23]. However, the photocatalytic efficiency of bare g-C_3_N_4_ is truncated by the high recombination rate of photogenerated charges (electrons and holes), insufficient light absorption and low specific surface area [24]. In this regard, various attempts have been made by researchers to eliminate these issues in recent times. Several strategies such as synthesis of mesoporous g-C_3_N_4_ [25], synthesis of 2D nanosheets [26], coupling with conductive materials [23], heterostructure formation with other semiconductor materials [27–28] and synthesis of carbon nitride like materials [29] have been explored to improve its photocatalytic activity.

Another attractive strategy to enhance the photocatalytic activity and to reduce the electron–hole recombination in a photocatalyst involves the coupling of a wide band gap material with a low band gap material [30]. Natarajan et al. have demonstrated the enhanced degradation of isoniazid (a pharmaceutical pollutant) over the g-C_3_N_4_–TiO_2_ nanocomposite via a direct Z-scheme charge transfer mechanism [31]. The enhanced photocatalytic activity has been attributed to the exact positions of energy band offsets of the coupled materials, which facilitates the photogenerated charge transfer and effectively suppresses their recombination. Moreover, perovskite materials are also potential candidates for environmental remediation applications and are well-explored in the literature [32–33]. Xian and coworkers have studied the photocatalytic degradation of MO under simulated solar light irradiation in BaTiO_3_–g-C_3_N_4_ composites with an efficient charge separation of photogenerated charge carriers at the interfaces [30]. Leong et al. have successfully made a promising amalgamated g-C_3_N_4_–SrTiO_3_ photocatalyst by a simple thermal method. The as-prepared composites showed exceptional properties for photocatalytic degradation of bisphenol A (BPA) under intense sunlight due to the enhanced migration of photogenerated charges over the close interfacial connections between g-C_3_N_4_ and SrTiO_3_ [34]. Thus, wide band gap materials can play an important role in maximizing the photocatalytic activity of g-C_3_N_4_ by suppressing the photogenerated charge recombination.

CaTiO_3_ (CT) is a well-known titanium-based perovskite material with a wide band gap of ≈3.5 eV and its activity is limited to UV excitation only [35]. Recently, our group has reported a novel RGO–N–CaTiO_3_ (RGO-NCT) bifunctional photocatalyst which comprises both adsorption and photocatalytic properties [36]. The photocatalytic activity of RGO-NCT photocatalysts was evaluated by studying the degradation of methylene blue (MB) and thiabendazole (TBZ) under visible light irradiation. Another attractive strategy for enhancement of photocatalytic performance is the coupling of a wide band gap material with a low band gap material, which allows the charge transfer via suitably arranged band edge positions in both materials [37]. Thus, the fabrication of a binary heterojunction of CT with a narrow band gap semiconductor like g-C_3_N_4_ can enhance the photocatalytic activity by effective charge separation and transfer across the heterojunction. Moreover, a sheet-like morphology can promote the photocatalytic activity, as such materials possess a large surface area, providing abundant active sites for reaction, and the short bulk diffusion length reduces the probability of recombination of the photogenerated charges. Herein, we report the optimized synthesis of sheet-like 2D CT nanoflakes for the first time via the polyacrylamide gel route. Furthermore, we made a promising 2D–2D heterojunction of CT nanoflakes and g-C_3_N_4_ nanosheets by a facile mixing method. The photocatalytic performance of the as-prepared CT–g-C_3_N_4_ (CTCN) heterojunction photocatalyst and the controls was evaluated by monitoring the degradation of rhodamine B (RhB) dye under UV, visible and natural sunlight irradiation. Also, the degradation of a non-photosensitizing colorless pollutant, BPA, was also studied under sunlight to eliminate the doubt of RhB photosensitization in the photocatalytic activity. The CTCN heterojunction offers enhanced light absorption both in the UV and visible light regions, leading to the formation of a higher number of charge carriers, their suppressed recombination, efficient separation and transfer over layered interface. Thus, we anticipate that the CTCN heterojunction can be used for the efficient removal of detrimental pollutants from water very effectively.

## Experimental

### Materials

Both titanium diisopropoxide bis(acetylacetonate) and dicyandiamide were purchased from Sigma-Aldrich, India. Calcium nitrate (Ca(NO_3_)_2_·4H_2_O), acrylamide and D-glucose were supplied by MP Biomedicals, India. Ammonia solution (NH_3_ about 25%), nitric acid (HNO_3_) and tartaric acid were supplied by Merck, India. All chemicals were used as received. Deionized (DI) water obtained from a double-stage water purifier (ELGA PURELAB Option-R7) was used for all experimental work.

### Synthesis of CaTiO_3_ nanoflakes

CaTiO_3_ nanoflakes were synthesized by adopting a reported procedure with some modifications [38]. In brief, equimolar ratio of Ca(NO_3_)_2_·4H_2_O and titanium diisopropoxide bis(acetylacetonate) (0.0075 mol) were dissolved in 20 mL of dilute HNO_3_ aqueous solution with a concentration of 1.6 mol L^−1^. To this solution, 0.0225 mol of tartaric acid, 20 g of glucose and 0.135 mol of acrylamide were successively added with constant stirring. The thus-obtained viscous solution was made up to 100 mL by adding distilled water, and aqueous ammonia was added dropwise to get a final pH of 2. This solution was heated at 70 °C on a hot plate to initiate the polymerization reaction and was kept at 120 °C for 24 h in an oven to obtain a black mass. The obtained xerogel was grounded into a powder and calcinated at 700 °C for 8 h, finally yielding white-colored CT nanoflakes.

### Synthesis of g-C_3_N_4_ nanosheets

The g-C_3_N_4_ nanosheets were synthesized by heating dicyandiamide at 550 °C with a temperature ramp of 3 °C/min for 4 h in static air and allowed to cool naturally to room temperature. The resultant yellow mass was well ground to form a fine powder. The thus-obtained powder was subjected to thermal oxidation etching in static air at 500 °C for 2 h with a ramp rate of 5 °C/min to get a pale yellow powder of g-C_3_N_4_ nanosheets upon cooling [16,39].

### Synthesis of CaTiO_3_–g–C_3_N_4_ 2D–2D heterojunction

The binary heterojunction photocatalyst (CTCN) was prepared by mixing both the organic (g-C_3_N_4_) and inorganic (CaTiO_3_) semiconductor materials in a 1:1 ratio by using a modified facile heating method [34]. In the typical procedure, 100 mg of as-prepared g-C_3_N_4_ nanosheets was ultrasonically dispersed in 20 mL of DI water. A similar dispersion of 100 mg of CT nanoflakes was also prepared in 20 mL of DI water and both solutions were mixed and stirred for 1 h. The final mixture was subjected to heating at 80 °C with constant stirring to get a thick suspension. The resulting suspension was then washed with DI water, centrifuged and dried overnight at 70 °C. The resultant material was finally ground into a fine powder employing a mortar and pestle and used for further experiments.

### Characterization

The purity, phase composition and structure of the resultant samples were identified by utilizing powder X-ray diffraction (XRD) studies using a Rigaku SmartLab 9 kW rotating anode diffractometer working in Bragg configuration with Ni-filtered Cu Ka irradiation (λ = 0.1542 nm) at 45 kV and 100 mA. The scans were collected over a 2θ range of 10–90° with a scan rate of 2° per minute. Fourier transform infrared (FTIR) spectra were recorded for functional group analysis by using an Agilent K8002AA Carry 660 instrument. Thermogravimetric analysis (TGA) was carried out in order to ascertain the stability of the as-prepared samples under nitrogen atmosphere by using a Netzsch STA 449 F1 Zupiter instrument. The samples were heated from room temperature to 800 °C at a heating rate of 10 °C min^−1^ with a flow rate of 40 mL min^−1^ in all experiments. Field-emission scanning electron microscopy (FE-SEM) was employed to explore the morphology and surface features of the resultant samples by using a FEI Nova Nano SEM-450 instrument. The samples were sputter-coated with a 2 nm Au layer before measurements in order to make them conducting. Energy dispersive X-ray spectroscopic analysis (EDAX) measurements were performed by using the same SEM instrument in order to find the elemental constituents of the samples. More detailed investigations on the morphology were obtained by transmission electron microscopy (TEM) and high-resolution TEM (HRTEM) studies. Images were recorded on a Technai G 20 (FEI) S-twin microscope operating at 200 kV (accelerating voltage). Elemental mapping was also carried out by using the same instrument in order to study the presence and spatial distribution of expected elements in all prepared samples. The light harvesting ability of the resultant samples as a dry-pressed disk was measured by diffuse reflectance spectroscopy (DRS). DRS studies were performed on a Perkin Elmer UV–visible–NIR Lambda 750 spectrophotometer by utilizing polytetrafluoroethylene (PTFE) polymer as a diffuse reflectance standard in the wavelength range 200–800 nm. Photoluminescence (PL) spectroscopy measurements were carried out by using an Agilent Technologies Cary Eclipse fluorescence spectrophotometer. The Brunauer–Emmett–Teller (BET) surface area and nitrogen adsorption–desorption isotherms were measured at 77 K on a Quantachrome Autosorb-iQ-MP-XR system.

### Photocatalytic degradation study

The evaluation of the photocatalytic performance of CTCN heterojunction and control samples was performed by monitoring the degradation of a model dye, RhB, which is a carcinogenic organic pollutant and is used illegally in the food industries in many countries [40]. The photocatalytic degradation ability tests were performed in a 50 mL glass conical flask by taking 25 mL of 1 × 10^−5^ M aqueous solution of RhB under UV light irradiation by using a Luzchem LZC 4V UV irradiation chamber equipped with 12 UV lamps (λ = 365 nm with intensity of approximately 32000 lux), under visible light irradiation by using a homemade photoreactor setup consisting of two 14 W white light LED bulbs (intensity approximately 55000 lux) and under intense sunlight on a full sunny day, separately. For each experiment, 25 mg of the photocatalyst was dispersed in 25 mL of aqueous solution of RhB and then stirred magnetically for 1 h in the dark to establish complete equilibrium of adsorption/desorption between the photocatalyst and the RhB dye before being subjected to irradiation. Blank experiments were also performed in the absence of any photocatalyst to observe the stability of RhB. During the photocatalytic reaction, 1 mL aliquots of the dispersion were extracted after 15 min regular intervals and centrifuged to separate the residual photocatalyst particulates. The photocatalytic degradation ability was examined by monitoring the change in the absorbance of the characteristic wavelength of RhB at 554 nm by using the UV–vis spectrophotometer. The photocatalytic degradation percentage (%) of the catalyst was calculated from the following relation [41–42]: Degradation (%) = (1 − *C*/*C*_0_) – 100, where *C*_0_ refers to the absorbance of RhB after adsorption equilibrium, achieved prior to the light irradiation, and *C* is the absorbance of RhB at the different time intervals under visible light illumination. For the BPA degradation study, it was dissolved in DI water with the help of probe sonication and the concentration of the final solution was kept at 5 × 10^−5^ M. Prior to irradiation, 25 mL of BPA solution mixed with 25 mg of CTCN heterojunction photocatalyst was kept in the dark for one hour under stirring conditions to attain adsorption–desorption equilibrium. Subsequently, the solution was kept under natural sunlight and aliquots of samples were collected at regular intervals of time by following the same procedure as performed in the case of RhB dye.

## Results and Discussion

### Synthesis and structural studies

**Figure 1:**
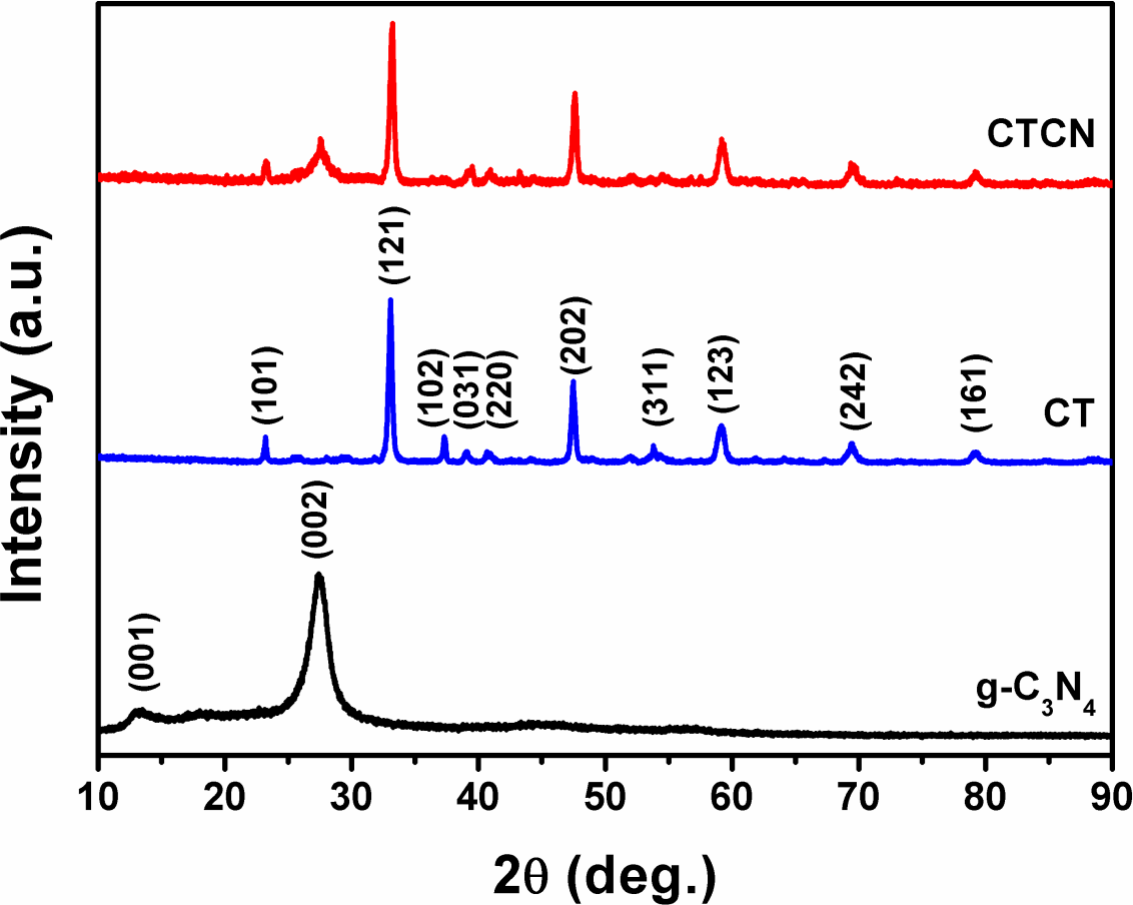
Powder X-ray diffraction patterns of g-C_3_N_4_, CT and CTCN heterojunction.

The heterojunction of g-C_3_N_4_ and CaTiO_3_ in a 1:1 ratio has been prepared by using a facile mixing method with well-defined 2D interfacial contacts. The powder XRD patterns of g-C_3_N_4_, CT and CTCN heterojunction were recorded to analyze the crystal phase structure and are presented in Figure 1. g-C_3_N_4_ nanosheets exhibit a weak diffraction peak at 2θ = 13.10^o^ (001) and an intense peak at 2θ = 27.34^o^ (002), which corresponds to the in-plane structural motif packing and interplanar stacking of aromatic systems, respectively, and are consistent with literature [16,34]. The diffraction pattern of CT nanoflakes shows peaks at 2θ = 23.20°, 33.05°, 37.30°, 39.08°, 40.68°, 47.49°, 53.78°, 59.18°, 69.43° and 79.20°, corresponding to the (101), (121), (102), (031), (220), (202), (311), (123), (242) and (161) planes, respectively, and are well indexed to the orthorhombic phase with high purity (JCPDS card no. 42−0423, *a* = 5.442 Å, *b* = 7.642 Å, *c* = 5.381 Å) [36]. All the peaks corresponding to CT could be identified in the powder XRD pattern of the CTCN heterojunction, illustrating the predominant presence and phase purity of CT. Although the (002) peak of g-C_3_N_4_ could be evidenced in the diffraction pattern of the CTCN heterojunction, the (001) peak of g-C_3_N_4_ could not be seen, which could be attributed to its weak diffraction intensity in comparison to the other peaks.

**Figure 2:**
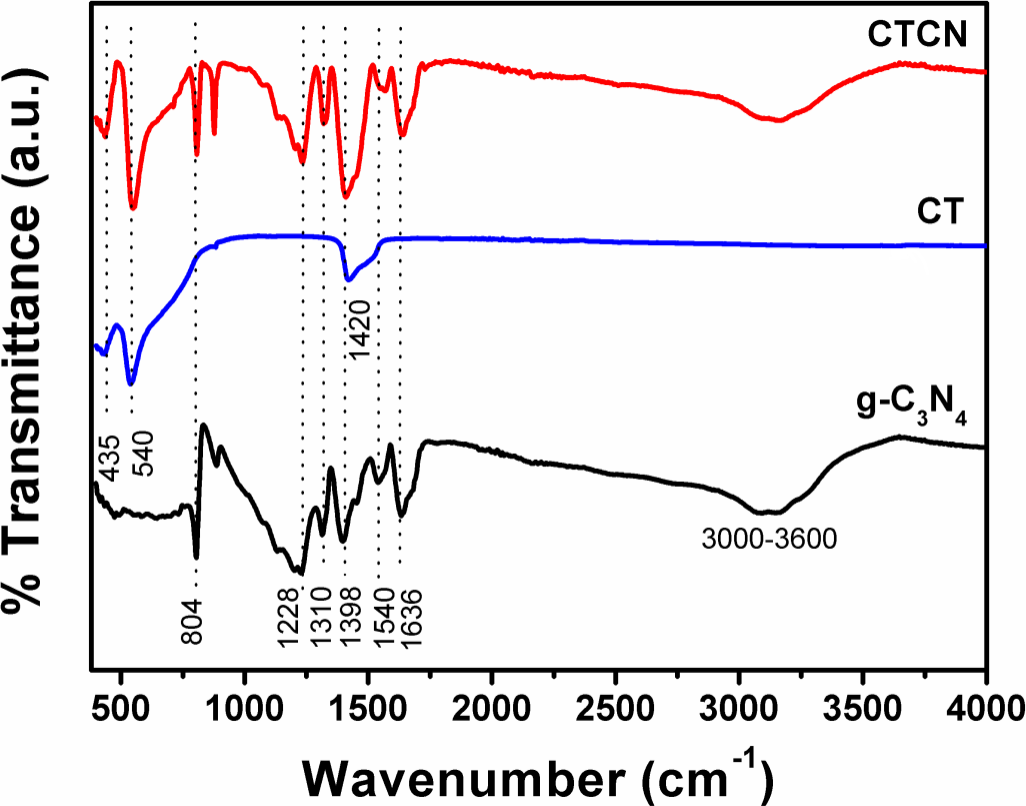
FTIR spectra of g-C_3_N_4_, CT and CTCN heterojunction.

Furthermore, FTIR spectroscopy has been employed for functional group analysis and the obtained spectra are shown in Figure 2. For g-C_3_N_4_, the sharp band around 807 cm^−1^ arises due to the presence of heptazine rings; the peaks at 1228 cm^−1^ and 1310 cm^−1^ can be assigned to the stretching vibrations of trigonal C–N(–C)–C or bridging C–NH–C units. The peaks at 1398 cm^−1^, 1540 cm^−1^ and 1636 cm^−1^ correspond to the stretching vibration of aromatic C–N bonds [16,34]. The broad peak from 3000–3600 cm^−1^ can be assigned to the terminal NH or NH_2_ groups of the aromatic rings and O–H stretching of surface hydroxyl groups [20]. The FTIR spectrum of CT nanoflakes shows three distinct peaks at 435 cm^−1^, 540 cm^−1^ and 1420 cm^−1^ corresponding to the stretching vibrations of Ti–O, bridging stretching modes of Ti–O–Ti and bending vibration of CO_3_^2−^ ions which is consistent with the literature [43]. The FTIR spectrum of the CTCN heterojunction comprises of all the peaks of g-C_3_N_4_ and CT, indicating that the addition of g-C_3_N_4_ has not altered the structural properties of CT, which is in agreement with the XRD results.

### Thermogravimetric studies

**Figure 3:**
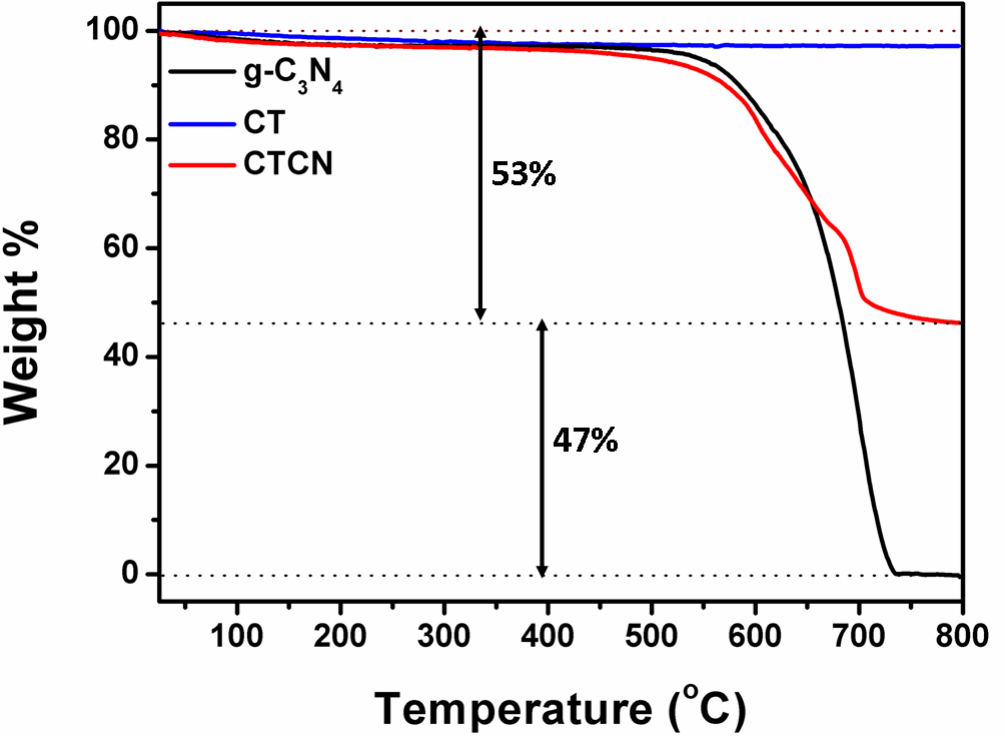
Thermogravimetric analysis plots of g-C_3_N_4_, CT and CTCN heterojunction.

To investigate the thermal stability of g-C_3_N_4_, CT and CTCN heterojunction, thermogravimetric (TGA) analysis was carried out from ambient temperature to 800 °C, at a heating rate of 10 °C min^−1^ under nitrogen atmosphere. The corresponding results are presented in Figure 3. For pure g-C_3_N_4_ nanosheets, the rapid decay of the TGA curve from 520 °C to 740 °C represents the typical thermal decomposition and indicates its intrinsic thermal instability in this temperature range [44]. CT nanoflakes show very high thermal stability in comparison to g-C_3_N_4_ and no significant weight loss is observed in the TGA curve of pure CT, even at 800 °C [36]. The relative content of the g-C_3_N_4_ in the CTCN heterojunction could also be verified from its TGA curve, which shows about 50% of each of the constituents, as expected from the preparation procedure. Overall, TGA analysis reveals that the as-prepared CTCN heterojunction possesses good thermal stability.

### Morphological and compositional studies

**Figure 4:**
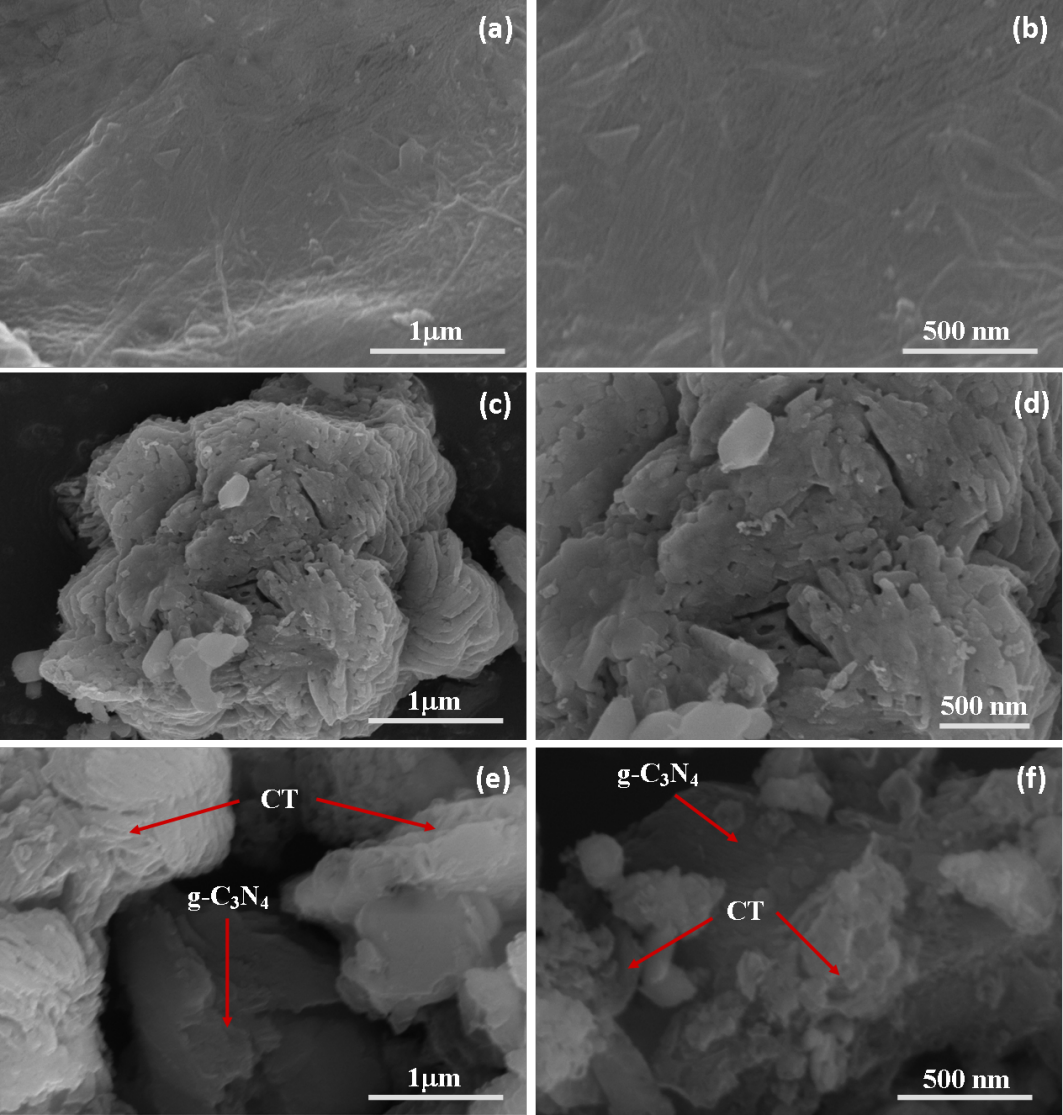
SEM images of (a, b) g-C_3_N_4_ sheets, (c, d) CT flakes and (e, f) CTCN heterojunctions.

The morphology of g-C_3_N_4_, CT and CTCN heterojunction were investigated by using SEM and TEM microscopic techniques. The SEM images of g-C_3_N_4_ are presented in Figure 4a and 4b, which shows an aggregated sheet-like morphology for bare g-C_3_N_4_. The SEM images of CT are presented in Figure 4c and 4d, which shows flake-like morphology. The SEM images of the CTCN heterojunction presented in Figure 4e and 4f shows the CT flakes deposited on g-C_3_N_4_ sheets, hence confirming the successful heterojunction formation with the 2D interface with face-to-face contact. The EDAX spectra of g-C_3_N_4_, CT and CTCN heterojunction presented in Figure S1, Supporting Information File 1 show the presence of all the constituent elements.

**Figure 5:**
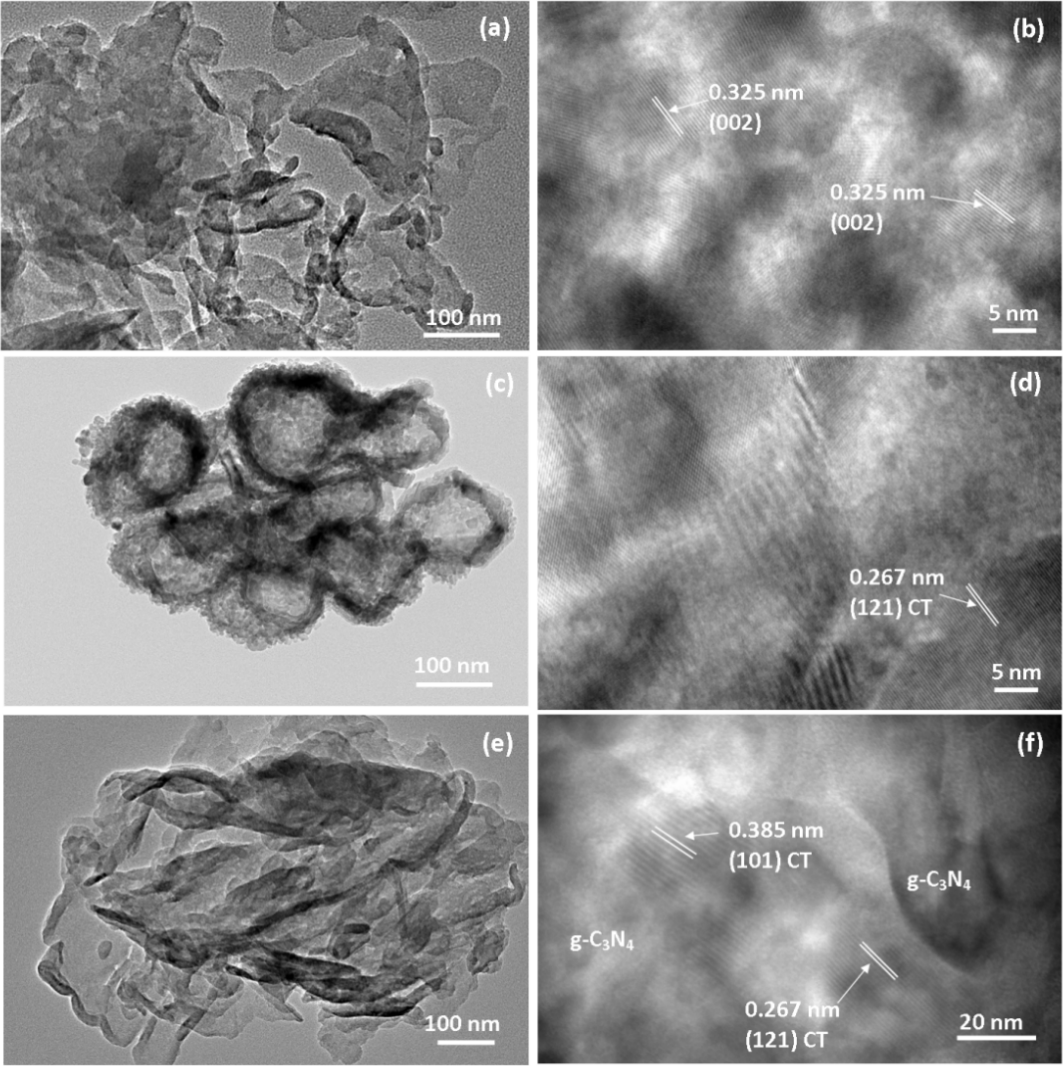
TEM images of (a, b) g-C_3_N_4_ nanosheets, (c, d) CT flakes and (e, f) CTCN heterojunction.

The TEM images of g-C_3_N_4_, CT and CTCN heterojunction are presented in Figure 5. Pure g-C_3_N_4_ exhibits a 2D lamellar sheet-like morphology (Figure 5a). The HRTEM image of g-C_3_N_4_ shows lattice fringes with 0.325 nm distance, which corresponds to its characteristic (002) plane (Figure 5b) [45]. The lattice fringes are not uniformly seen, which is due to the semi-crystalline nature of g-C_3_N_4_ as previously reported in the literature [46]. It can be seen from Figure 5c that CT has a thin sheet-like morphology joined together to form an aggregated flake-like structure. Furthermore, lattice fringes with 0.267 nm distance corresponding to the (121) plane of CT are observed in Figure 5d [47]. A TEM image of the CTCN sample presented in Figure 5e shows well-anchored CT nanoflakes on the g-C_3_N_4_ sheets, which form a heterojunction due to their close interfacial contact. The heterojunction formation can be confirmed in Figure 5f as the presence of CT is confirmed with 0.267 nm (121) and 0.385 nm (101) lattice fringes along with g-C_3_N_4_ sheets clearly marked [48]. The lattice fringes of g-C_3_N_4_ are not observed in the heterojunction, which could be due to its poor crystallinity [46]. This interfacial contact favors the charge transfer between CT and g-C_3_N_4_, which suppress the recombination of photogenerated charge carriers and enhance the photocatalytic activity. Furthermore, the elemental mapping of g-C3N4, CT and CTCN was also carried out in order to study the presence and spatial distribution of expected elements and corresponding maps are provided in Figure S2, S3 and S4, Supporting Information File 1, respectively. The mapping results show the uniform distribution and co-existence of expected elements in the selected area. Moreover, these results also show that the CTCN heterojunction is not simply a physical mixture of separate CT and g-C_3_N_4_ entities [49].

### Optical properties study

**Figure 6:**
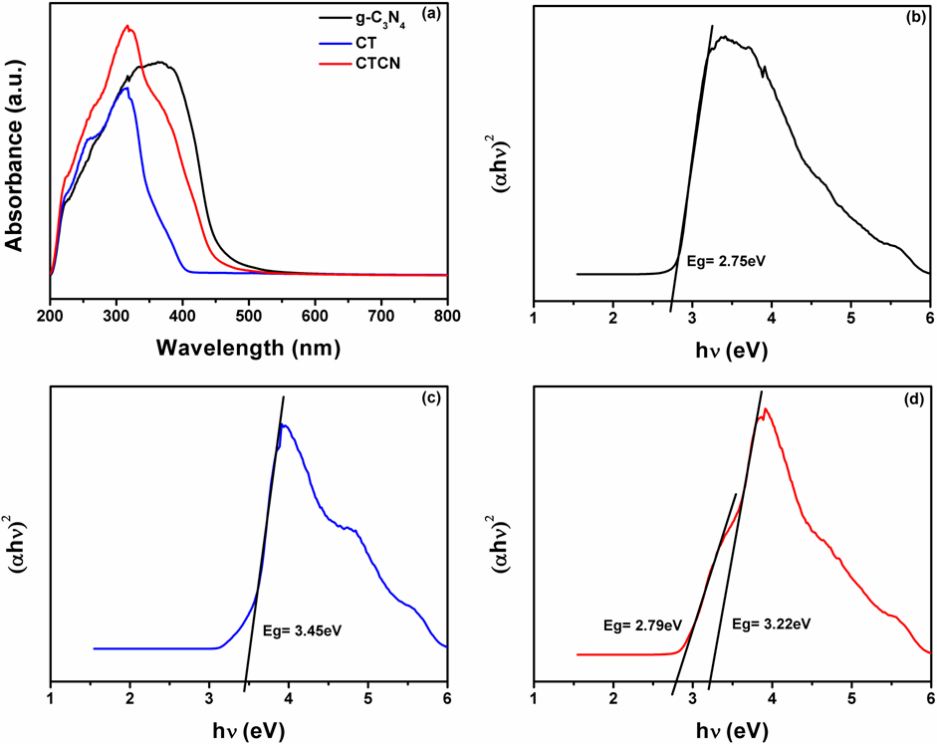
(a) UV–visible diffuse reflectance spectroscopy (DRS) spectra for g-C_3_N_4_, CT and CTCN heterojunction; Plot of transformed Kubelka–Munk function vs energy for (b) g-C_3_N_4_, (c) CT and (d) CTCN heterojunction.

The band gap energy of a material is directly related to its light harvesting capability. To gain insight into the band structure of heterojunction, a DRS study has been performed with all prepared catalysts. The DRS plots for g-C_3_N_4_, CT and CTCN heterojunction are depicted in Figure 6a. The band gap values of g-C_3_N_4_, CT and CTCN heterojunction are calculated from the transformed Kubelka–Munk plots (Figure 6b–d). Bare g-C_3_N_4_ nanosheets unveil a distinct absorption edge in the visible region corresponding to the band gap of 2.75 eV resulting from the transfer of electrons from the valence band to the conduction band [50]. The absorption edge for CT nanoflakes lies in the UV region exhibiting a band gap of 3.45 eV. The DRS profile of CTCN heterojunction presents a combination of the spectral features of g-C_3_N_4_ and CT, showing absorption both in the UV (3.22 eV) and visible regions (2.79 eV). The combination of these two materials may result in an effective photocatalyst that can absorb a larger part of the solar light spectrum, encompassing both the UV and visible light regions.

**Figure 7:**
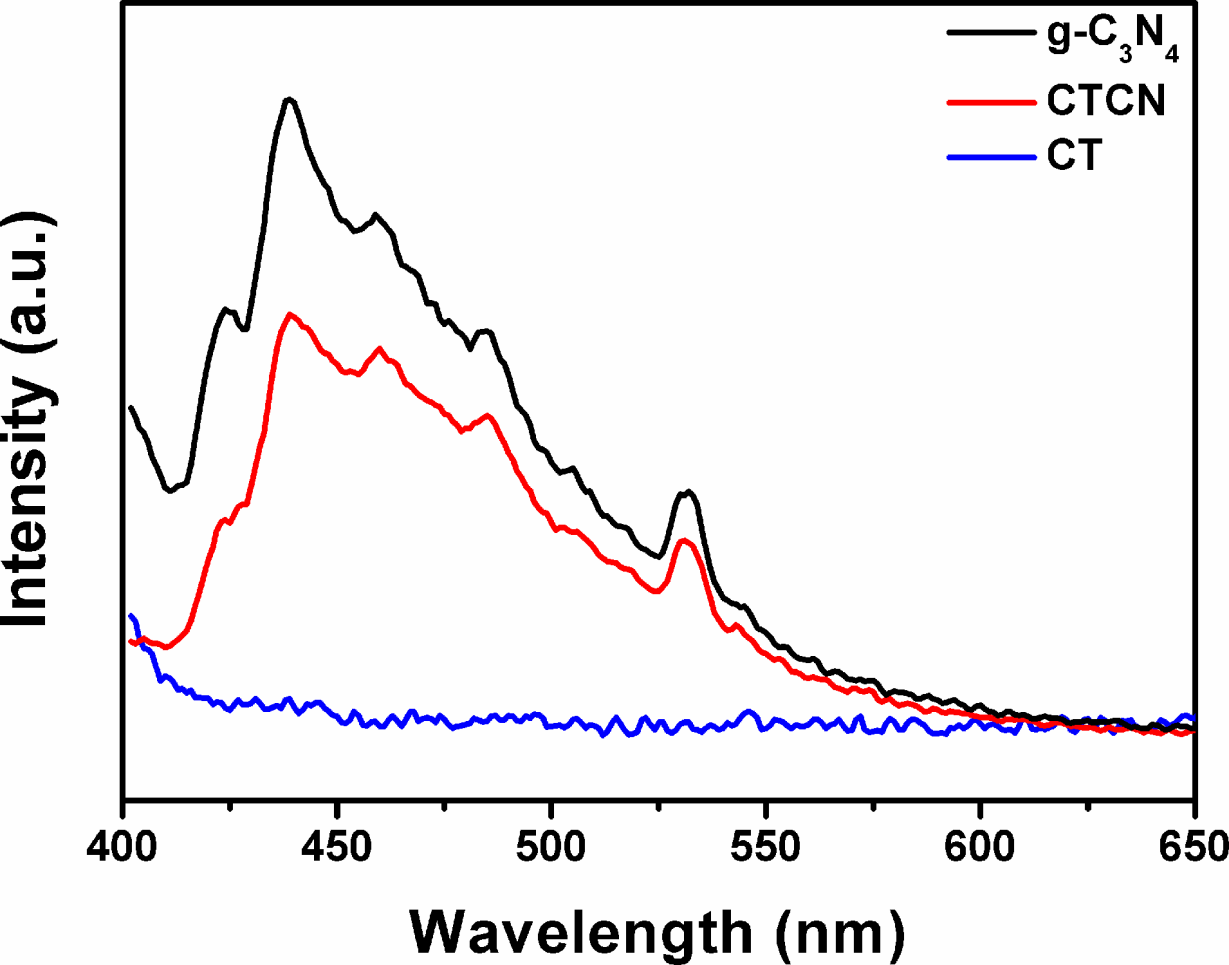
Photoluminescence spectra of g-C_3_N_4_, CT and CTCN heterojunction.

In addition, photoluminescence (PL) spectroscopy studies were carried out in order to study the separation and migration of photogenerated charge carriers in the CTCN heterojunction. It is well-reported in literature that recombination of electrons and holes give rise to intense PL emissions [51]. The g-C_3_N_4_ nanosheets show high PL emission, which is an indication of a high recombination rate of photogenerated charge carriers produced during light irradiation. However, the formation of the CTCN heterojunction allows the migration of photogenerated charges across the 2D–2D interface, which results in the prolongation of their lifetime. This is evident from the reduced PL emission intensity of CTCN heterojunction as compared to the bare g-C_3_N_4_ nanosheets as shown in Figure 7.

### Surface area studies

**Figure 8:**
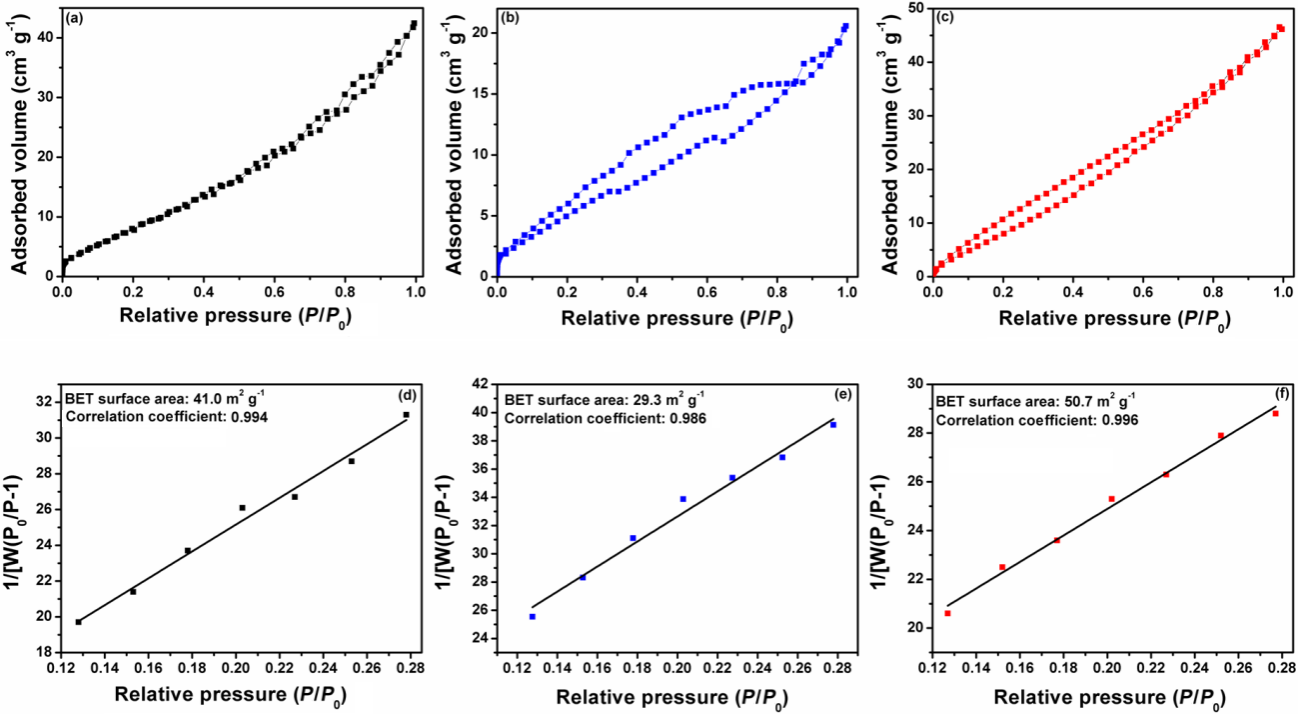
Nitrogen adsorption–desorption curves for (a) g-C_3_N_4_ (b) CT and (c) CTCN heterojunction; BET surface area plots for (d) g-C_3_N_4_ (e) CT and (f) CTCN heterojunction.

In order to explore the influence of g-C_3_N_4_ nanosheets on the surface area of CT flakes and to investigate the effect of g-C_3_N_4_ on enhancing the photocatalytic activity of the CTCN heterojunction, the BET surface area of bare CT, g-C_3_N_4_ and CTCN heterojunction has been investigated. Figure 8a–c represents the N_2_ adsorption–desorption isotherms for g-C_3_N_4_, CT and CTCN heterojunction, respectively. The corresponding BET surface area plots and values are presented in Figure 8d–f. It can be seen that the BET surface area of bare CT flakes was estimated to be 29.3 m^2^g^−1^ and the pure g-C_3_N_4_ nanosheets was 41.0 m^2^g^−1^. However, upon coupling of g-C_3_N_4_ nanosheets with CT flakes, the BET surface area of the resulting CTCN composite was enhanced to 50.7 m^2^g^−1^, which is larger in comparison to that of bare samples. This enhancement could be due to the exfoliation of g-C_3_N_4_ nanosheets and CT nanoflakes due to the ultrasonication process adopted in the synthesis of the CTCN heterojunction. Thus, the high surface area of CTCN heterojunction photocatalyst provides more reaction sites for the adsorption and degradation of pollutant molecule and hence results in the enhancement of photocatalytic activity.

### Photocatalytic activity studies

**Figure 9:**
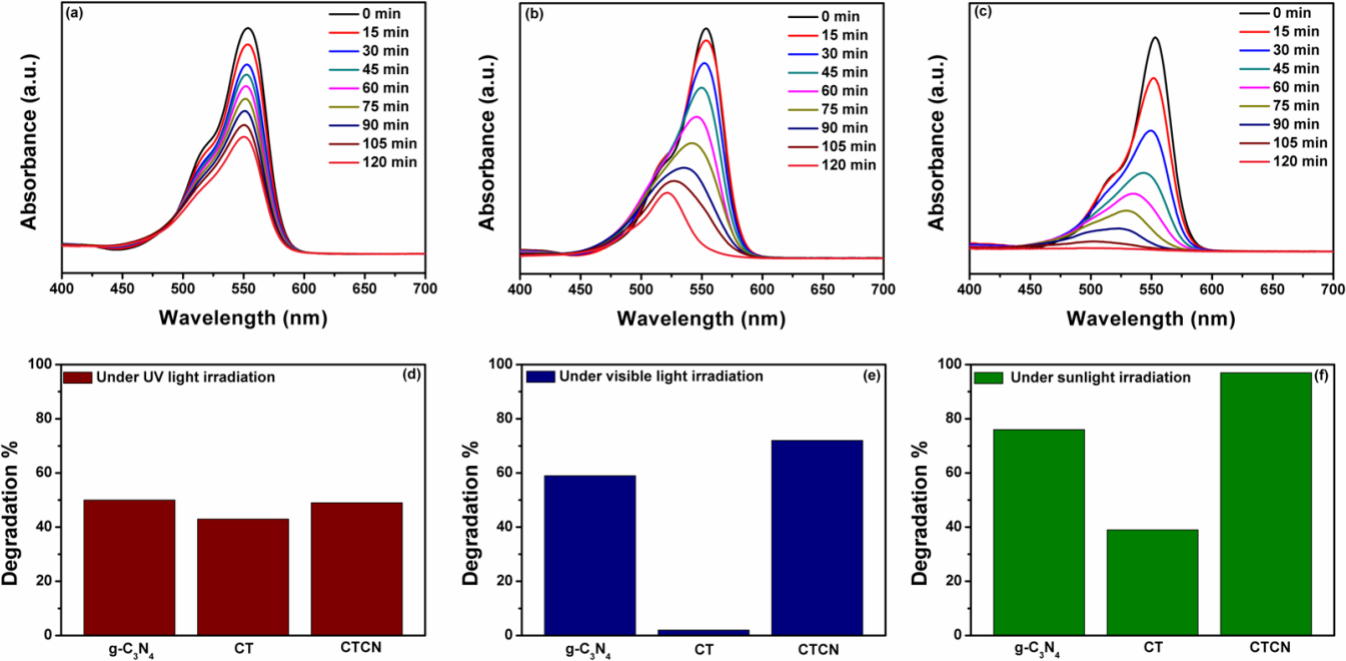
Time-dependent absorption spectra of RhB degradation with the CTCN heterojunction under (a) UV light, (b) visible light and (c) sunlight irradiation; degradation percentage plots of RhB with different samples under (d) UV light, (e) visible light and (f) sunlight irradiation after 120 min of irradiation.

The photocatalytic activity of the CTCN heterojunction and the corresponding control samples was investigated by studying the degradation of a model dye pollutant, rhodamine B (RhB) under UV, visible and natural sunlight irradiations. The prominent absorption peak of RhB at 554 nm was monitored by employing UV–vis spectroscopy to quantify the amount of dye degraded. The time-dependent absorption spectra of RhB solutions degraded by the CTCN heterojunction photocatalyst under different light sources are depicted in Figure 9a–c and those for bare g-C_3_N_4_ and CT, including the control experiments performed without catalyst, are provided in Figure S5, Supporting Information File 1. It can be seen from the absorption plots presented in Figure 9a–c and Figure S5a–c, Supporting Information File 1, that a shift in the absorption maximum of RhB along with the decrease in intensity is observed upon irradiation under any light source [52]. Generally, the degradation of RhB takes place either by destruction of the chromophore or by *N*-deethylation of RhB, resulting in the decrease in absorption at λ_max_ = 554 nm. *N*-Deethylation of RhB leads to a steady blue shift toward 495 nm with increase in the irradiation time in the presence of g-C_3_N_4_, which could be due to the formation of *N*-deethylated intermediates. However, direct chromophore destruction leads to the formation of low molecular weight fragments, which does not have absorption in the lower visible region [53]. In our study, we observed a distinguishable blue shift in the λ_max_ of RhB solution containing the bare g-C_3_N_4_ or CTCN heterojunction, under all three types of irradiation. Thus, we can say that degradation of RhB takes place via *N*-deethylation processes in this case. However, in case of bare CT nanoflakes, no blue shift was observed in the λ_max_ which infers direct destruction of the chromophore of RHB during the degradation process. Control experiments for the degradation of RhB without any catalyst reflect its high stability under different light irradiation conditions, as no significant decrease in the absorption was observed. Figure 9d–f represents the degradation percentage of RhB upon irradiation under different light sources. It can be seen from Figure 8d that in the presence of UV light irradiation, bare g-C_3_N_4_ degrades ≈50% of the RhB dye. This degradation is obvious because high-energy UV light can also cause excitation of electrons from the valence band to the conduction band of g-C_3_N_4_. In the presence of bare CT nanoflakes, ≈43% degradation of RhB could be achieved. The CTCN heterojunction also degraded only ≈50% of RhB, indicating that it is only as good as the bare g-C_3_N_4_ under UV irradiation. However, under visible light irradiation (Figure 8e), the CTCN heterojunction performed better by degrading ≈72% of RhB, while bare g-C_3_N_4_ could degrade only ≈59% of RhB. On the other hand, the RhB degradation achieved using bare CT nanoflakes was almost negligible as visible light did not have any effect on this wide band gap material. In addition, another set of control experiments performed on a simple physical mixture of CT and g-C_3_N_4_ showed substantially lower photocatalytic activity compared to the CTCN heterojunction. This indicates that the coupling of CT with g-C_3_N_4_ results in the formation of a heterojunction between them and enhances the photocatalytic performance under visible-light irradiation. The CTCN heterojunction provides a platform for the quick transfer of the photogenerated charge carriers from g-C_3_N_4_ to CT, thereby preventing their recombination and increasing their lifetime. This heterojunction formation also played a crucial role under natural sunlight irradiation (Figure 9f), wherein the CTCN heterojunction showed ≈97% degradation of RhB, much higher than bare g-C_3_N_4_ (≈76%) and CT (≈39%). The enhanced photocatalytic activity observed for a CTCN heterojunction under sunlight irradiation as compared to UV and visible light irradiation could be attributed to the following three reasons: (i) extended light absorption by the CTCN heterojunction both in the UV and visible region of solar energy, (ii) greater number of photoinduced charge carriers generation due to g-C_3_N_4_ and CT excitation by sunlight and (iii) fast charge transfer across a 2D interface to produce active species, which causes mineralization of pollutants. Therefore, our photocatalytic activity studies illustrate that the CTCN heterojunction formed by coupling CT with g-C_3_N_4_ performs well in comparison to its bare counterparts under all three irradiation conditions (i.e., UV, visible and sunlight).

To demonstrate the photocatalytic performance of the as-prepared catalysts more precisely, the kinetics of the photodegradation of RhB aqueous solutions was studied by fitting the obtained degradation results to a pseudo-first-order reaction model [54–55] and modified Freundlich model [56–57], utilizing the following integral equation respectively:


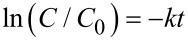



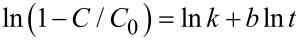


**Figure 10:**
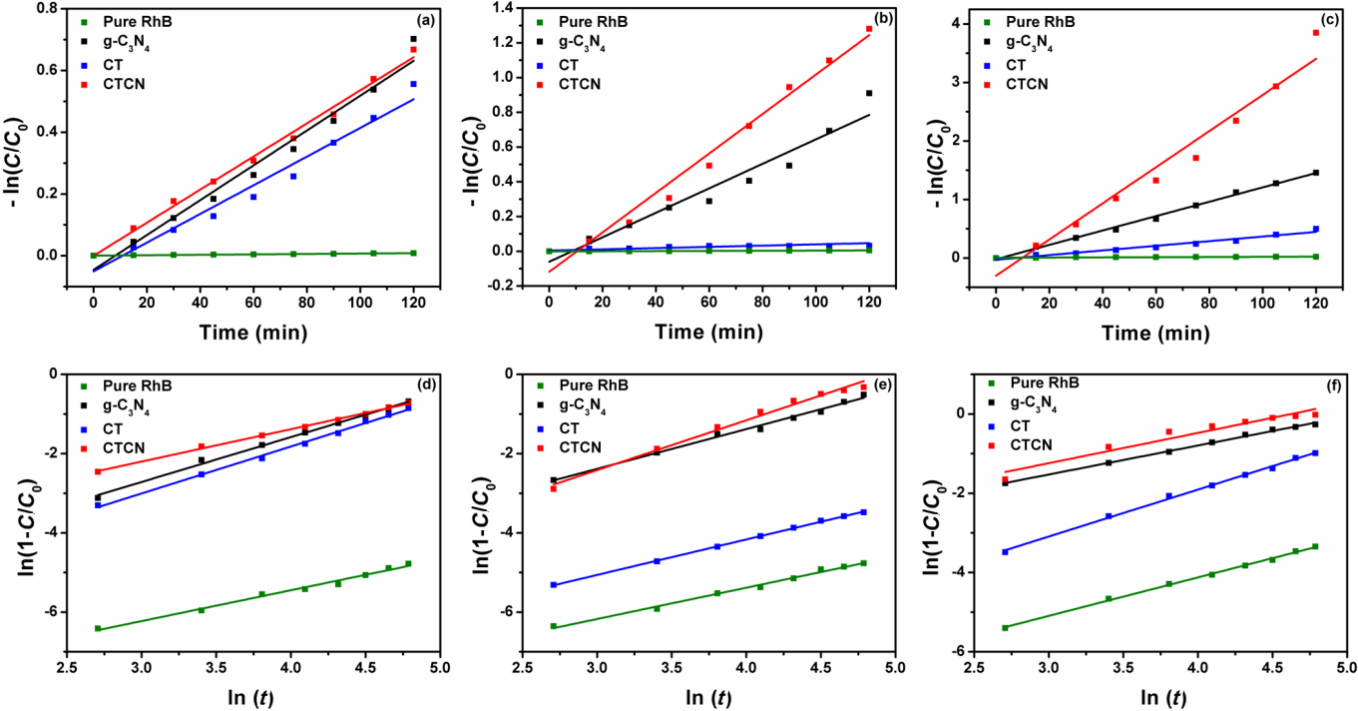
Kinetic curves obtained by applying (a, b, c) pseudo-first-order and (d, e, f) the modified Freundlich model for RhB degradation with different photocatalysts under (a, d) UV light, (b, e) visible light and (c, f) sunlight irradiation.

In these equations, *C*_0_ is the absorbance of RhB after attaining the adsorption equilibrium, achieved prior to light illumination, *C* is the absorbance of RhB at time interval *t* under light illumination and *k* is the degradation rate constant. The kinetic plots corresponding to a pseudo-first-order reaction model are presented in Figure S6, Supporting Information File 1. Figure S6a–c represents the *C*/*C*_0_ vs time plots for the catalysts under UV, visible and sunlight irradiation, respectively. The corresponding logarithmic plots are presented in Figure 10a–c under UV, visible and sunlight irradiation, respectively, according to the pseudo-first-order reaction model. The degradation rate constants (*k*) acquired from the slope of the plot of −ln(*C*/*C*_0_) vs time are presented in Table S1 (Supporting Information File 1) along with corresponding linear regression coefficients (R^2^). The kinetic plots obtained by fitting the experimental data to the modified Freundlich model are presented in Figure 10d–f. The calculated *k* and R^2^ values are provided in Table S2 in Supporting Information File 1. From the analysis of the kinetic plots and corresponding R^2^ values obtained from both kinetic models, it can be concluded that degradation of RhB follows the modified Freundlich model more appropriately. The removal of RhB from its aqueous solution is controlled by adsorption of the dye molecules on the photocatalyst surface followed by their successive degradation as per the modified Freundlich model [56]. Therefore, the photocatalytic degradation reaction happens on the surface of the photocatalysts only. Also, the analysis of the degradation constants suggests that the CTCN heterojunction offers the best degradation of RhB under different light irradiation conditions, with sunlight showing the highest activity.

**Figure 11:**
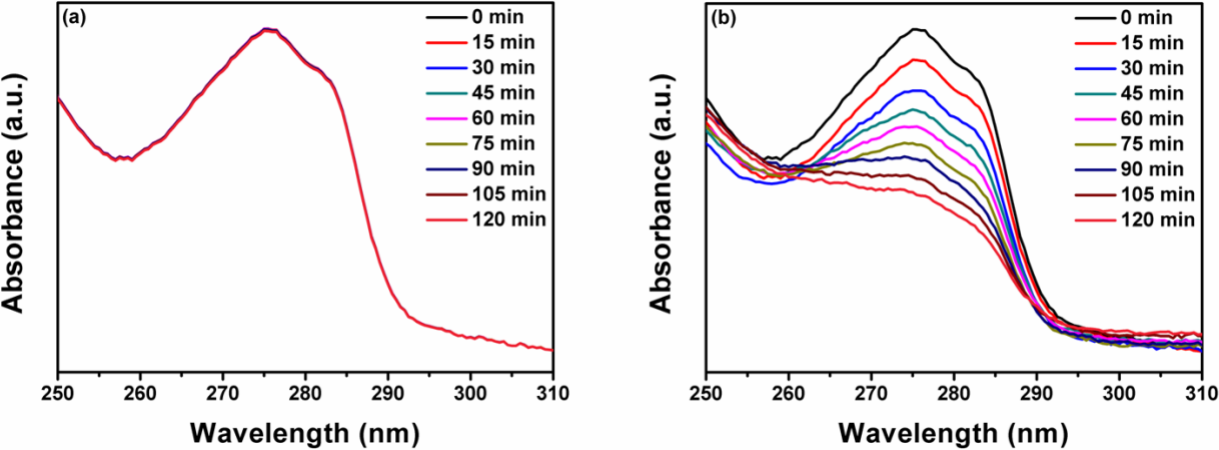
Time-dependent absorption spectra of BPA degradation under sunlight irradiation (a) pure BPA (without catalyst) and (b) using CTCN heterojunction.

Furthermore, we have also studied the degradation of a non-photosensitizing colorless pollutant, BPA, against the CTCN heterojunction under natural sunlight irradiation. BPA is an industrially important compound used in the production of polycarbonates and other plastics. It comes under the class of endocrine disruptors and is known to mainly causes damage to the reproductive system and fertility in human beings [58]. The absorption peak at 275 nm was monitored as presented in the degradation plot of BPA shown in Figure 11. After 120 min of irradiation under sunlight ≈47% degradation of BPA could be observed using the CTCN heterojunction as catalyst, while the control experiments performed without any catalyst showed no degradation. Thus, it can be concluded that the as-prepared heterojunction photocatalyst has favorable solar light harvesting capability and can be utilized for the degradation of various water pollutants. We have also quantified the adsorption of both RhB and BPA over different prepared photocatalysts. The adsorption percentage of both the pollutants was very low, which infers that the photocatalytic action is solely due to the degradation of adsorbed molecules (Figure S7, Supporting Information File 1). From Figure S7a, it can be seen that the difference in the absorbance of pure RhB solution and after attaining adsorption–desorption equilibrium is much less, which corresponds to about 6% adsorption of dye molecules on the surface of the CTCN heterojunction, while for BPA, only 7% adsorption was observed over the CTCN heterojunction, as depicted in Figure S7b, Supporting Information File 1.

### Photocatalyst reusability studies

**Figure 12:**
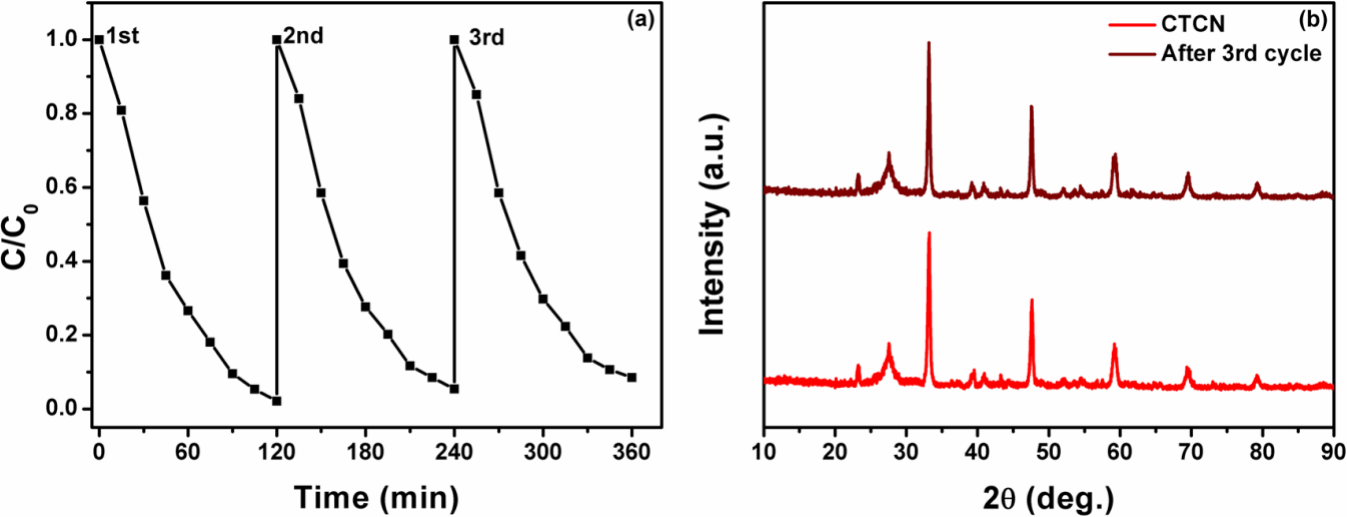
(a) Photocatalyst reusability up to three cycles and (b) powder XRD pattern of a CTCN heterojunction before and after the third cycle.

It is of immense importance to explore the photostability and recyclability of the photocatalyst materials as it could appreciably reduce the costs of the photocatalytic process and reveals the most promising photocatalysis candidates. Hence, we carried out three successive cycles of photodegradation of RhB in order to comment on the reusability of the CTCN heterojunction as an efficient photocatalyst. The results obtained are presented in Figure 12a. Analysis of the recyclability results shows only an indiscernible decrease (≈7%) in the photocatalytic performance of the CTCN heterojunction, even after three successive cycles under natural sunlight illumination, which could be due to the unavoidable catalyst loss in the recycling process. Powder XRD spectra of the as-prepared and recycled CTCN heterojunction are also presented in Figure 12b. It can be seen from the PXRD pattern that there is no apparent change in the structure of the catalyst following the three successive cycles of photocatalytic degradation. Thus, the recycling results reflect the commendable stability of the CTCN heterojunction and support its potential for environmental remediation applications.

### Mechanism of photocatalytic activity

**Figure 13:**
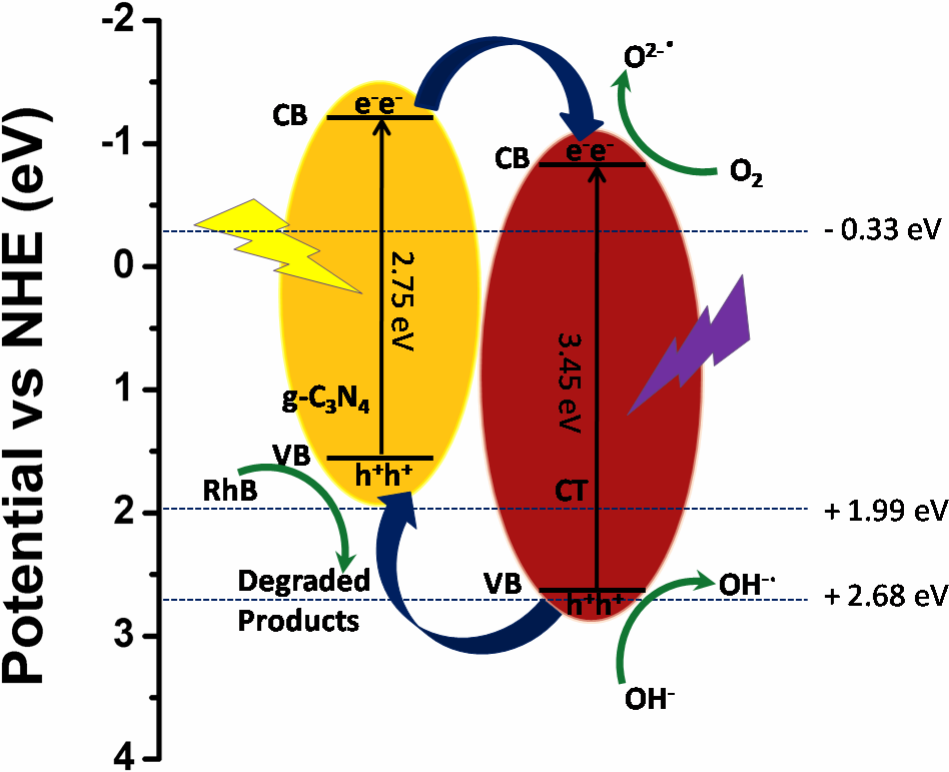
Plausible mechanism of degradation of pollutants under sunlight irradiation using the CTCN heterojunction photocatalyst.

The attainment of high efficiency of charge separation during a photocatalytic reaction plays an important role in the significant enhancement of the photocatalytic activity of heterojunction photocatalysts. However, the separation of photoinduced charge carriers totally relies on the appropriate band edge positions of the two constituent materials of the heterojunction photocatalyst, which are responsible for the separation and migration of photogenerated charges. The appropriate band positions of the semiconductor materials produce space charge accumulation/depletion at the interfaces, which helps in the effective separation of photogenerated charge carriers [59]. In this regard, the valence band (VB) and conduction band (CB) edge potentials of g-C_3_N_4_ and CT can be calculated by using following equations [36,54]:


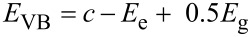



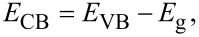


where *E*_VB_* and E*_CB_ are the VB and CB edge potentials and *E*_g_ is the bandgap of the semiconductor materials. The band gap values for g-C_3_N_4_ and CT are 2.75 eV and 3.45 eV respectively, obtained from DRS measurements. χ is the Sanderson electronegativity with values 4.64 eV and 5.40 eV for g-C_3_N_4_ and CT, respectively [34,36]. *E*_e_ is the energy of free electrons on the hydrogen scale (4.5 eV vs NHE). Based on this information, the calculated *E*_CB_ values for g-C_3_N_4_ and CT are −1.23 eV and −0.83 eV vs NHE, respectively, and *E*_VB_ values are 1.52 eV and 2.62 eV vs NHE, respectively. The appropriate band gap positions and close interfacial contact between g-C_3_N_4_ nanosheets and CT nanoflakes inhibit the fast recombination of electron–hole pairs, resulting in an enhanced photocatalytic activity for the degradation of the pollutant (RhB and BPA in this case). Based on these facts, a reasonable mechanism for the enhanced photocatalytic activity of the CTCN heterojunction under sunlight irradiation has been proposed as schematically presented in Figure 13, which involves the promotion of the charge transfer efficiency at the g-C_3_N_4_-CT interface.

In the case of the bare photocatalysts, CT (being UV light active, 3.45 eV) can undergo excitation under sunlight irradiation to generate electrons and holes. Also, g-C_3_N_4_ (2.75 eV) absorbs in the sunlight to induce photogenerated electron–hole pairs. Unfortunately, due to the fast recombination of photogenerated charges, a small fraction of them implicated in the photocatalytic degradation process which leads to the relatively low activity of the g-C_3_N_4_. In addition, CT could not be excited under visible light irradiation because of its wide band gap and it exhibits a weaker photocatalytic activity. When CT is coupled with g-C_3_N_4_, the formation of an effective heterojunction (CTCN) takes place, which facilitates the transfer of photogenerated electrons from the CB of g-C_3_N_4_ to that of CT through the intimate interface as governed by their band edge positions and hinders the charge recombination. This electron transfer is thermodynamically highly favored as charge flows from higher negative potential value to the lower negative potential values [34,60]. Thus, the formation of O_2_^−•^ radicals is facilitated here as the reduction potential of O_2_/O_2_^−^ (−0.33 eV vs NHE) is less negative in comparison with the CB edge potential of CT (−0.83 eV). Therefore, the transferred electrons react with O_2_ to reduce it into O_2_^−•^ radical anions. In comparison to the standard redox potentials of ^•^OH/H_2_O (2.68 eV vs NHE) and ^•^OH/OH^−^ (1.99 eV vs NHE), the h^+^ species left on the VB of g-C_3_N_4_ cannot react with OH^−^ or H_2_O to produce ^•^OH radicals due to the more negative VB potential of g-C_3_N_4_ (1.52 eV vs NHE). However, the h^+^ on the VB of CT (2.62 eV) can react with the OH^−^ ions to generate ^•^OH radicals due to more positive potential as compared to OH^−^/^•^OH (1.99 eV vs NHE) but cannot react with H_2_O directly according to the band potential values (^•^OH/H_2_O = 2.68 eV vs NHE) [54]. Also the migration of h^+^ from the VB of CT to the VB of g-C_3_N_4_ is also possible as holes can migrate upwards [61–62]. Therefore, it can be concluded that O_2_^−•^, h^+^ and ^•^OH species participate in the photodegradation of the pollutant. The entire photocatalytic reaction mechanism under sunlight irradiation can be summarized as follows:


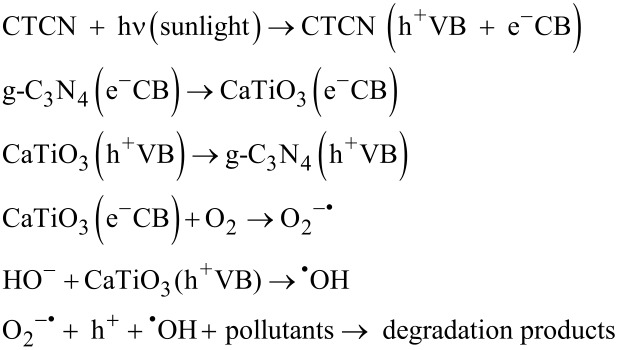


**Figure 14:**
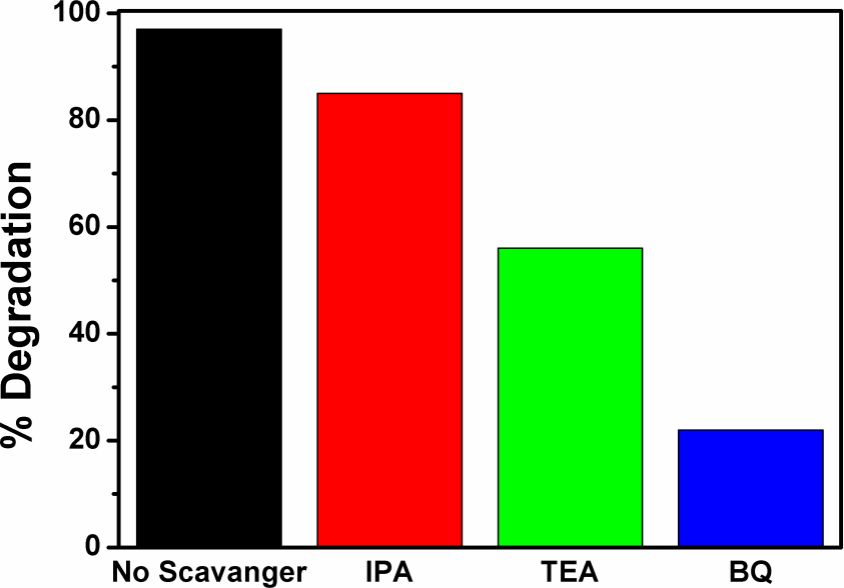
Effect of scavengers on the photocatalytic degradation of RhB using the CTCN heterojunction photocatalyst under natural sunlight illumination.

To validate the proposed mechanism and to gain insight into the role played by active species, radical trapping experiments were performed for RhB degradation under natural sunlight illumination using the CTCN heterojunction as the photocatalyst. In this study, benzoquinone (BQ), isopropanol (IPA) and triethanolamine (TEA) were used as scavengers for superoxide radicals (O_2_^−•^), hydroxyl radicals (^•^OH) and photoexcited holes (h^+^), respectively, and the corresponding results are depicted in Figure 14 [54]. It can be seen from the Figure 14 that the photodegradation of RhB solution was drastically quenched in the presence of BQ showing the dominance of O_2_^−•^ radical anions in the degradation process. Also, a significant decrease in degradation percentage is also observed in the presence of TEA pointing towards the active role of h^+^ species in the degradation of RhB. However, ^•^OH radicals do not contribute much as the addition of IPA and had only a small effect on the degradation process of RhB. Thus, the trapping experiment results also confirm that O_2_^−•^ and h^+^ were the dominant reactive species and the involvement of ^•^OH was less important in the decomposition of RhB.

The above proposed mechanism holds true for UV light irradiation as well, as per our experimental observations, because g-C_3_N_4_ also shows significant degradation of RhB under UV light irradiation, which cannot be attributed to the dye photosensitization alone. High energy UV radiation can also cause excitation of electrons from the VB of g-C_3_N_4_ to its CB. However, this mechanism may not be applicable for visible light irradiation, as CT does not absorb at all in the visible region and excitation of electrons from the VB of CT to the CB is not possible. The role of each of the semiconductor material (CT and g-C_3_N_4_) in the heterojunction is clearly elucidated by these experiments.

## Conclusion

In conclusion, a promising CTCN heterojunction photocatalyst with intimate interfacial contact was successfully prepared by a facile low temperature mixing of g-C_3_N_4_ nanosheets and CT nanoflakes in a 1:1 ratio. The photocatalytic activity of the CTCN heterojunction photocatalyst was investigated by studying the degradation of an aqueous solution of RhB under UV, visible and sunlight irradiation conditions. The prepared CTCN heterojunction exhibited the highest photocatalytic activity in comparison to the bare g-C_3_N_4_ and CT samples. The prepared CTCN heterojunction has also been proven effective to degrade a non-photosensitizing, colorless pollutant, bisphenol A, under sunlight irradiation. The enhanced photocatalytic performance could be attributed to the appropriate band positions, close interfacial contact between g-C_3_N_4_ nanosheets and CT nanoflakes, excellent heterojunction formation and high specific surface area, which provides larger surface active sites for the reaction and facilitates the transfer of photogenerated charges across the heterojunction by inhibiting their fast recombination. Superoxide radicals (O_2_^−•^) were found to contribute as the main active species in trapping experiments for the decomposition of RhB under sunlight irradiation. A plausible mechanism for the enhanced photocatalytic activity of the CTCN heterojunction photocatalyst has been proposed based on our experimental results and theoretically calculated band potential values. Therefore, in our understanding, this study can provide insights into the design, development and mechanistic study of two-dimensional heterojunction photocatalysts with enhanced photocatalytic activity.

## Supporting Information

File 1 Additional experimental results.
EDAX spectra of g-C_3_N_4_, CT and CTCN heterojunction (Figure S1). Elemental mapping of g-C_3_N_4_, CT and CTCN composite (Figure S2–S4). Time-dependent absorption spectra of RhB degradation under different light irradiation conditions for control samples and pure RhB (Figure S5). *C*/*C*_0_ vs time plots for the photocatalytic degradation of RhB under UV, visible and sunlight irradiation (Figure S6). Summary of the kinetic data of photocatalytic degradation of RhB (Table S1 and S2). Absorption curves for the calculation of adsorption percentage of RhB and BPA (Figure S7).
