# Supplementary material for: Perovskite-structured CaTiO3 coupled with g-C3N4 as a heterojunction photocatalyst for organic pollutant degradation
Source: Beilstein J Nanotechnol. 2018 Feb 21;9:671–85. doi: 10.3762/bjnano.9.62 (PMC5827633; doi:10.3762/bjnano.9.62)
Supplement: File 1 — Additional experimental results. EDAX spectra of g-C3N4, CT and CTCN heterojunction (Figure S1). Elemental mapping of g-C3N4, CT and CTCN composite (Figure S2–S4). Time-dependent absorption spectra of RhB degradation under different light irradiation conditions for control samples and pure RhB (Figure S5). C/C0 vs time plots for the photocatalytic degradation of RhB under UV, visible and sunlight irradiation (Figure S6). Summary of the kinetic data of photocatalytic degradation of RhB (Table S1 and S2). Absorption curves for the calculation of adsorption percentage of RhB and BPA (Figure S7). [file Beilstein_J_Nanotechnol-09-671-s001.pdf]

# Perovskite-structured $CaTiO_3$ coupled with g-$C_3N_4$ as a heterojunction photocatalyst for organic pollutant degradation

Ashish Kumar, Christian Schuerings, Suneel Kumar, Ajay Kumar and Venkata Krishnan*

Address: School of Basic Sciences and Advanced Materials Research Center, Indian Institute of Technology Mandi, Kamand, Mandi 175005, Himachal Pradesh, India

Email: Venkata Krishnan - vkn@iitmandi.ac.in

*Corresponding author

# Additional experimental results

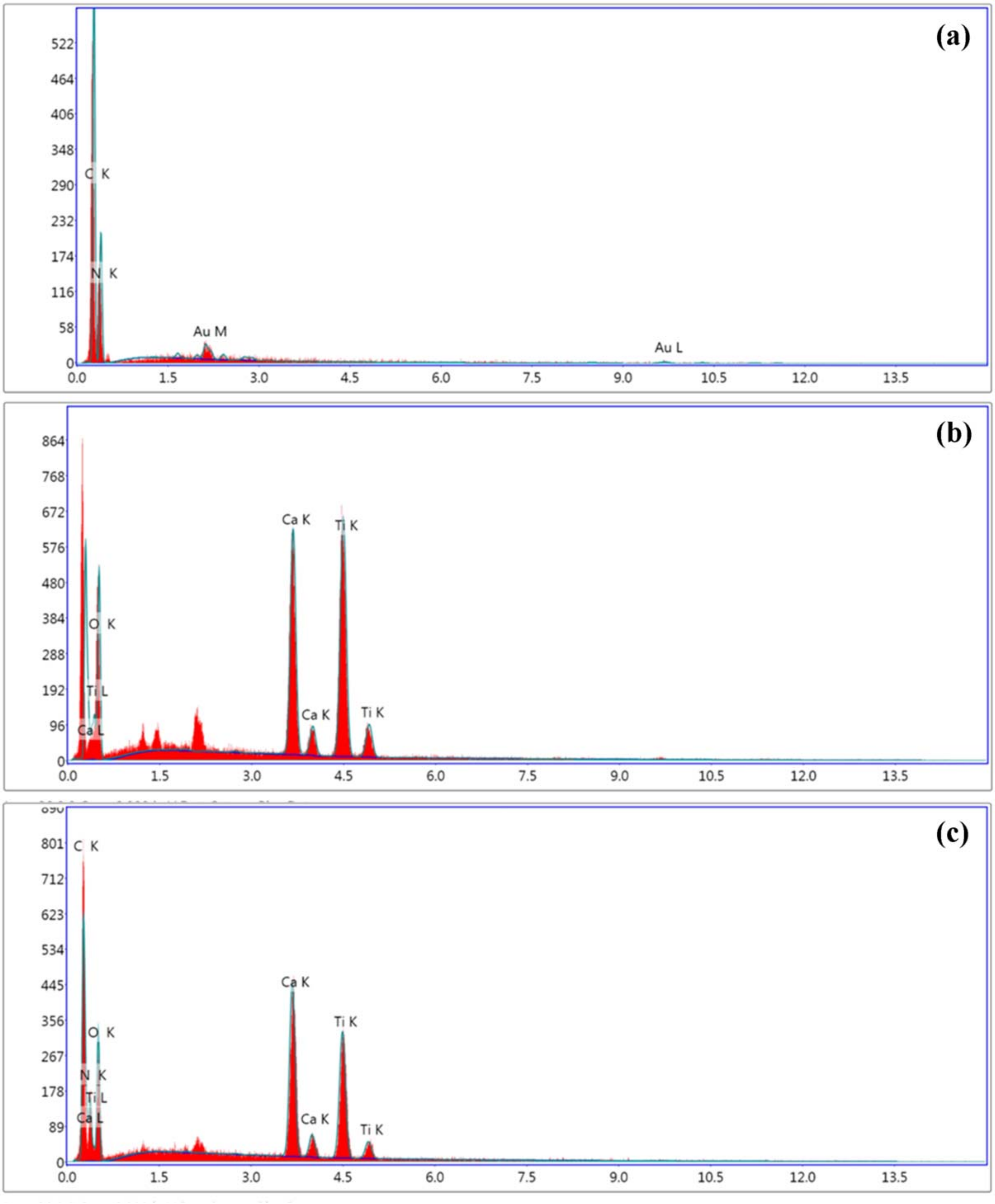


**Figure S1.** EDAX spectra of (a) g-$C_3N_4$, (b) CT and (c) CTCN heterojunction.

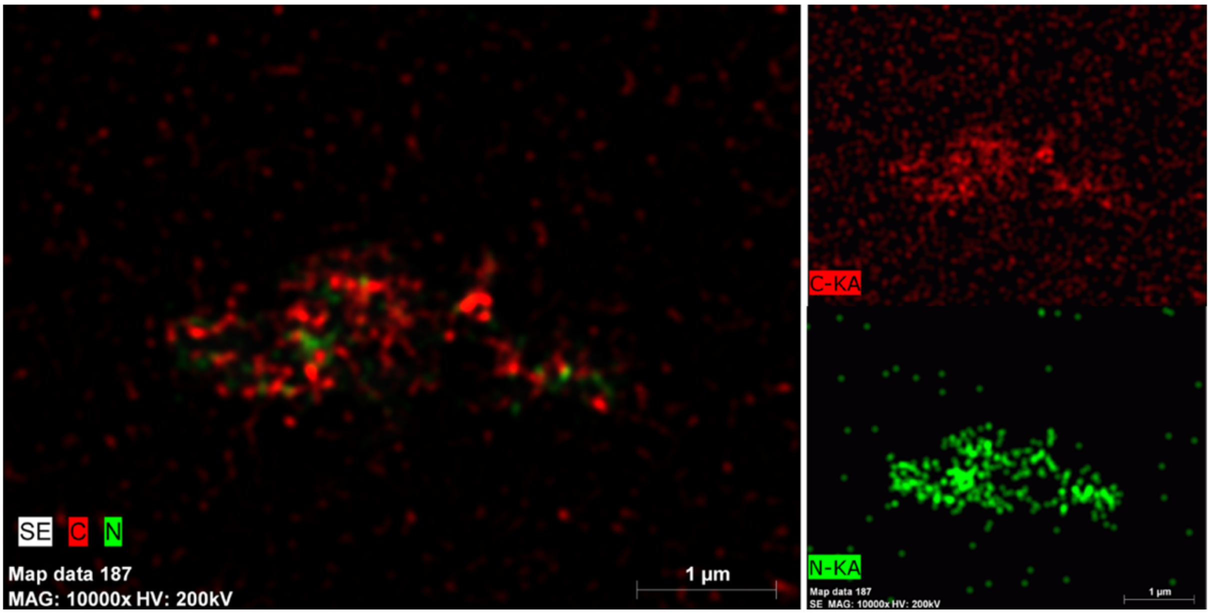


**Figure S2.** Elemental mapping of  g-$C_3N_4$ sheets.

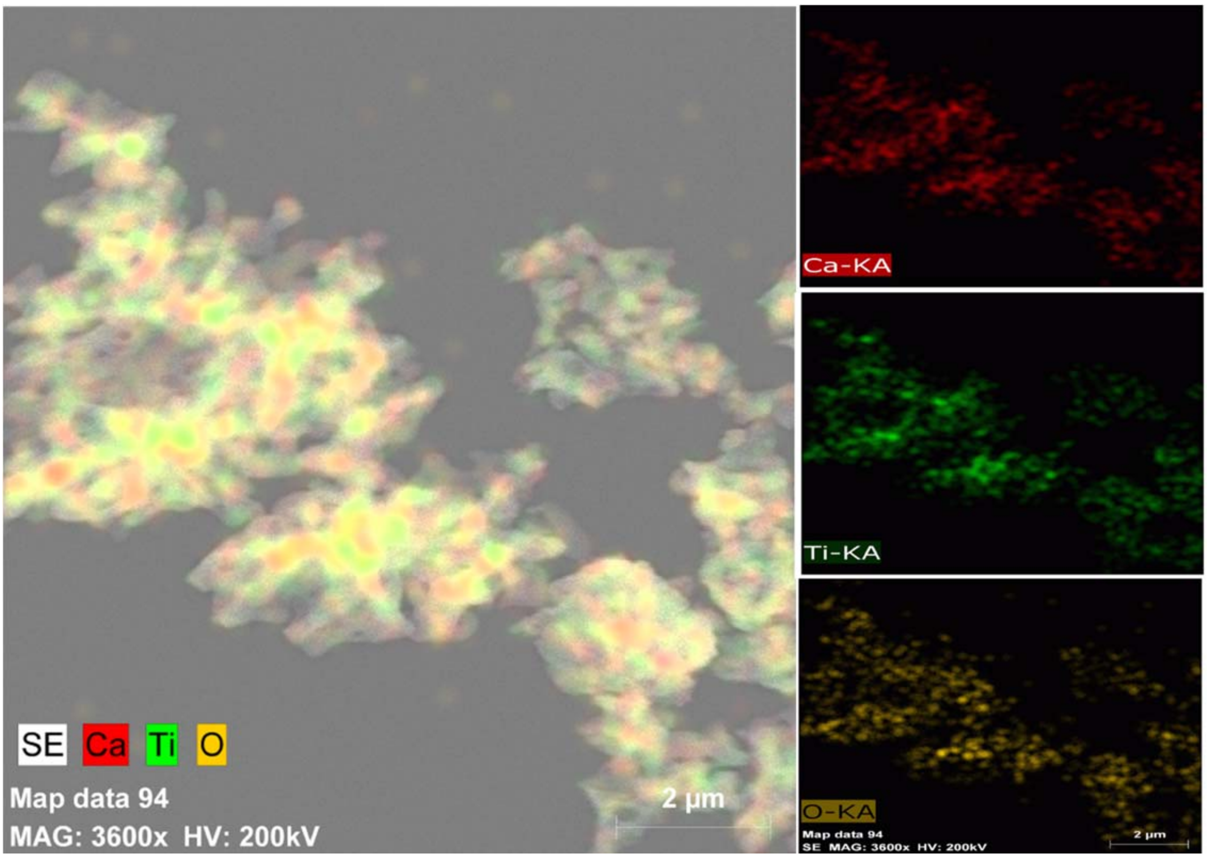


**Figure S3.** Elemental mapping of  CT nanoflakes.

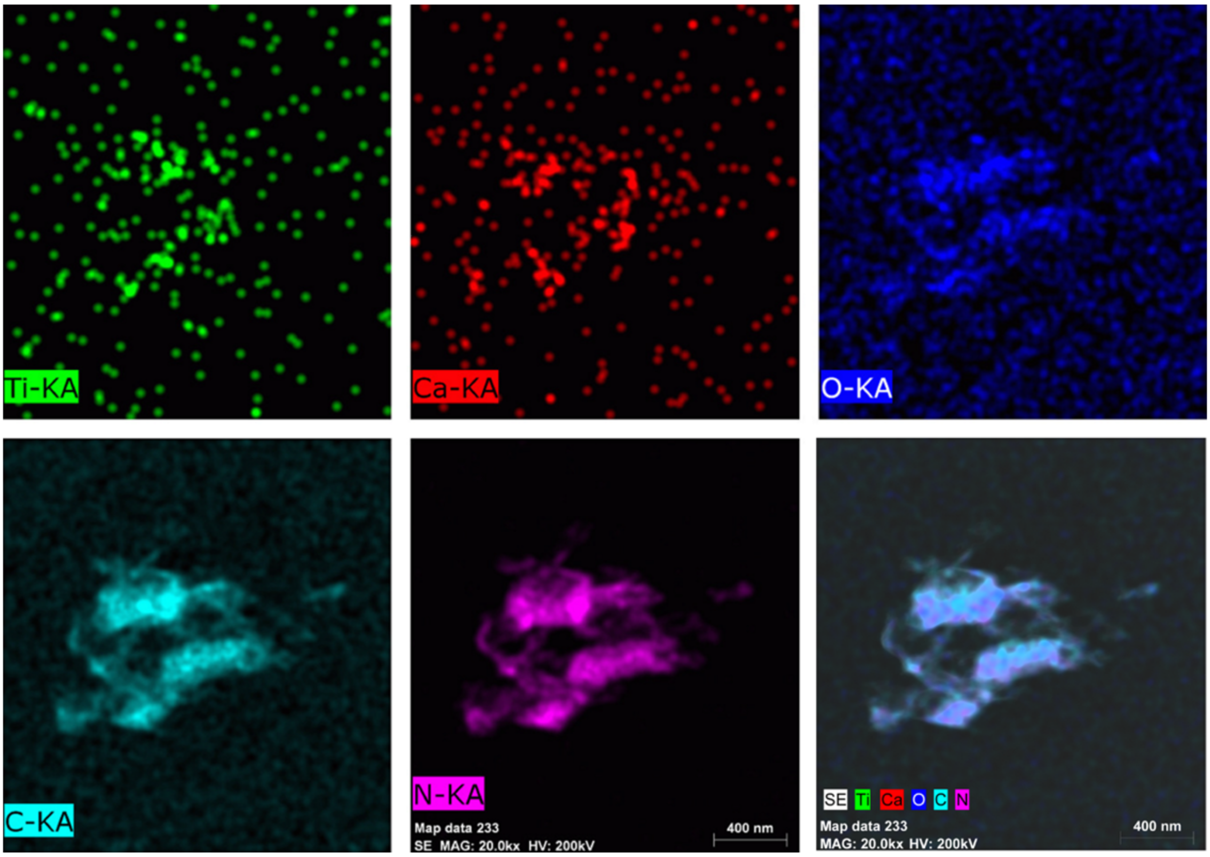


**Figure S4.** Elemental mapping of CTCN hetrojunction.

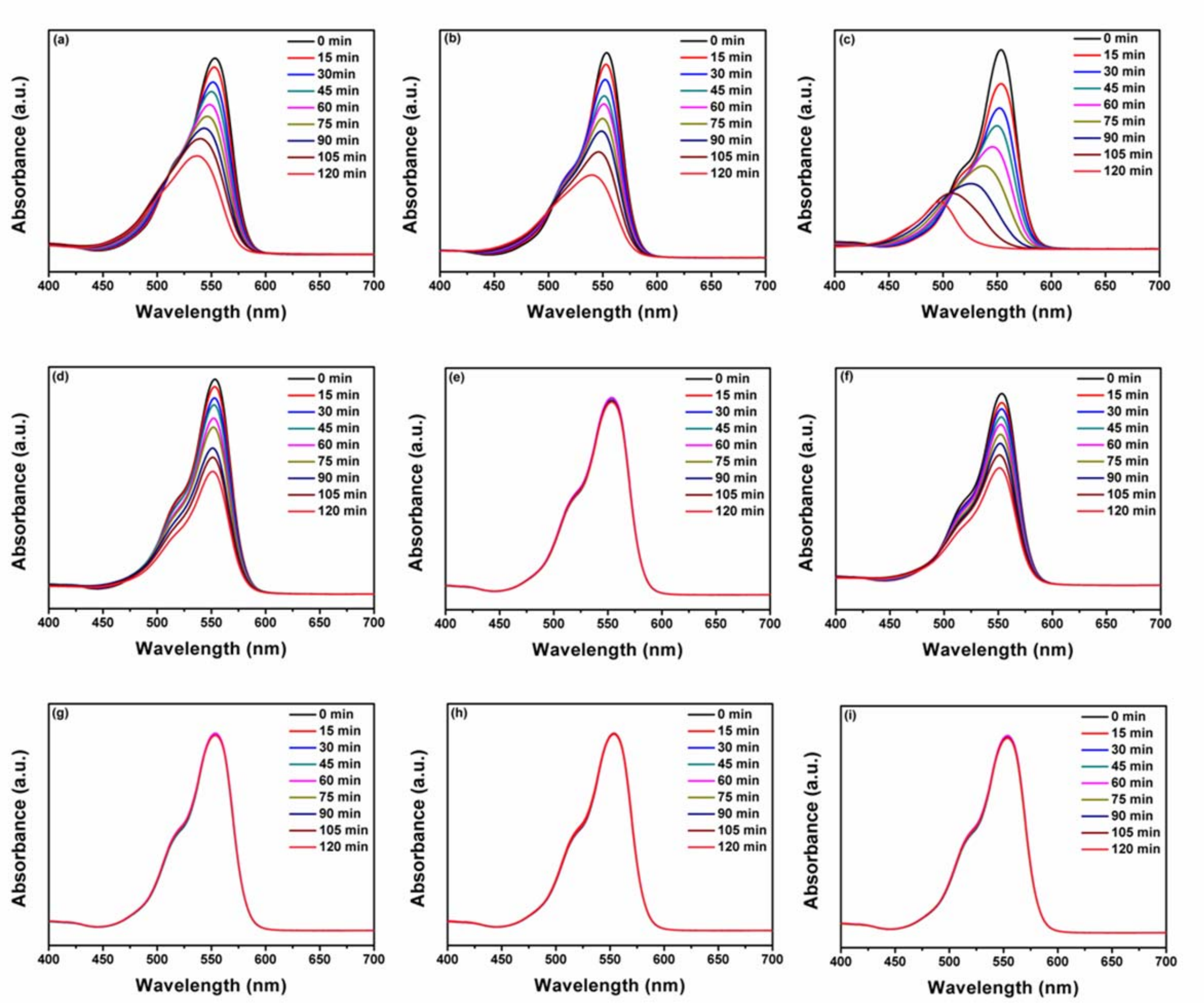


**Figure S5.**Time-dependent absorption spectra of RhB degradation over (a, b, c) g-$C_3N_4$, (d, e, f) CT and (g, h, i) pure RhB (without catalyst) under UV, visible and sunlight irradiations, respectively.

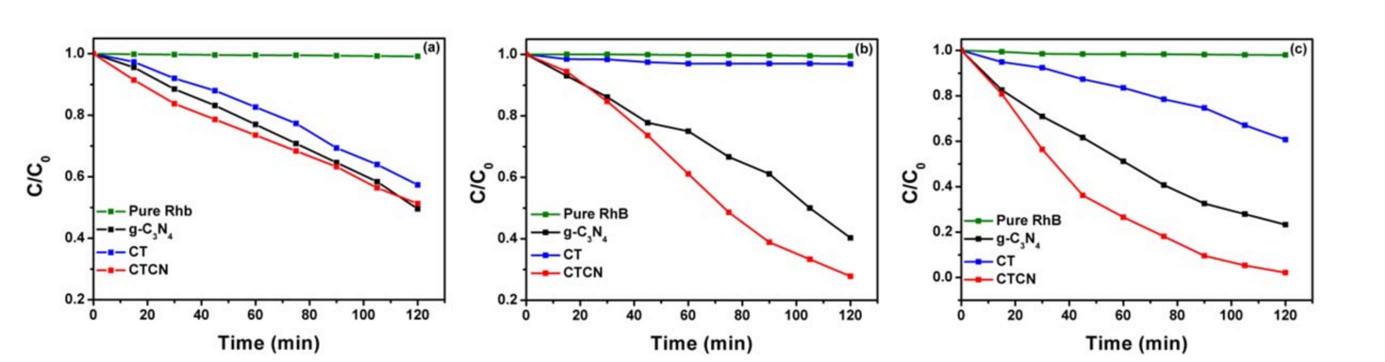


**Figure S6.** $C/C_0$ vs time plots for the photocatalytic degradation of RhB under UV, visible and sunlight irradiation**.**

**Table S1.** Summary of kinetic data obtained by applying pseudo first order kinetic model for the photocatalytic degradation of RhB using as prepared photocatalysts under different light irradiations.

| **Under UV light irradiation** | | |
|---|---|---|
| **Composite** | **Rate constant *k*** | **$R^2$** |
| g-$C_3N_4$ | 5.6 X $10^{-3}$ | 0.97 |
| CT | 4.6 X $10^{-3}$ | 0.97 |
| CTCN | 5.4 X $10^{-3}$ | 0.99 |
| **Under visible light irradiation** | | |
| g-$C_3N_4$ | 7.0 X $10^{-3}$ | 0.94 |
| CT | 0.4 X $10^{-3}$ | 0.98 |
| CTCN | 11.4 X $10^{-3}$ | 0.98 |
| **Under sunlight irradiation** | | |
| g-$C_3N_4$ | 12.3 X $10^{-3}$ | 0.99 |
| CT | 4.0 X $10^{-3}$ | 0.96 |
| CTCN | 30.9 X $10^{-3}$ | 0.96 |

**Table S2.** Summary of kinetic data obtained by applying modified Freundlich model for the photocatalytic degradation of RhB using as prepared photocatalysts under different light irradiations.

| Under UV light irradiation | | |
|---|---|---|
| **Composite** | **Rate constant *k*** | **$R^2$** |
| g-$C_3N_4$ | 2.2 X $10^{-3}$ | 0.99 |
| CT | 1.4 X $10^{-3}$ | 0.99 |
| CTCN | 9.4 X $10^{-3}$ | 0.99 |
| **Under visible light irradiation** | | |
| g-$C_3N_4$ | 4.6 X $10^{-3}$ | 0.99 |
| CT | 0.4 X $10^{-3}$ | 0.97 |
| CTCN | 2.1 X $10^{-3}$ | 0.99 |
| **Under sunlight irradiation** | | |
| g-$C_3N_4$ | 24.1 X $10^{-3}$ | 0.99 |
| CT | 1.3 X $10^{-3}$ | 0.98 |
| CTCN | 29.2 X $10^{-3}$ | 0.96 |

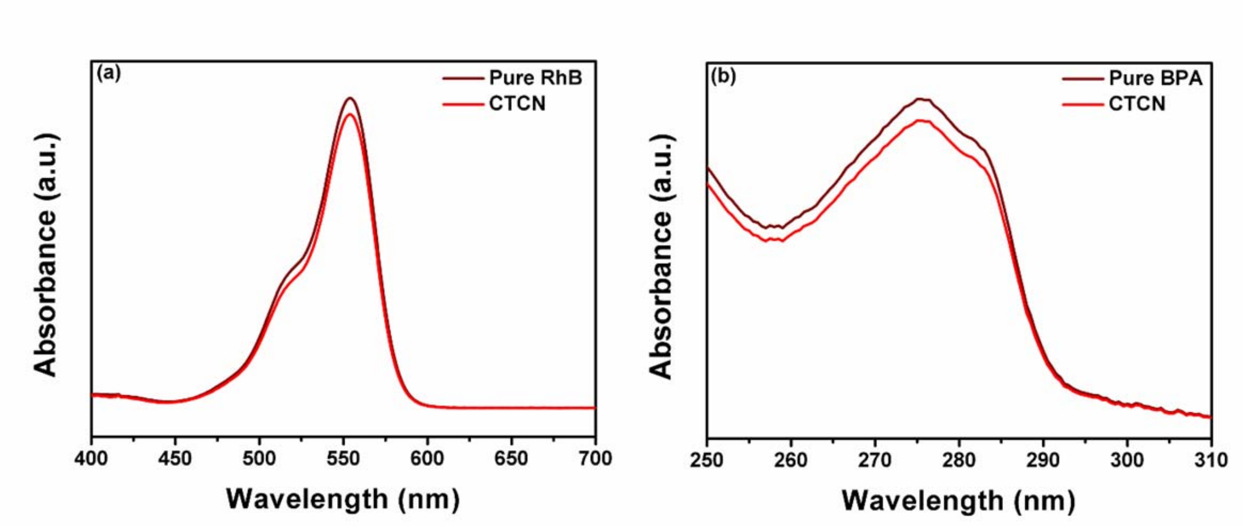


**Figure S7.** Absorption spectra for calculation of adsorption percentage of (a) RhB over CTCN and (b) BPA over CTCN heterojunction.
